# Effects of a community pharmacy cardiovascular practice transformation (CPT) program on blood pressure

**DOI:** 10.1016/j.rcsop.2024.100559

**Published:** 2024-12-25

**Authors:** William R. Doucette, Eilan Alhersh, Lindsey Ludwig, Stevie Veach

**Affiliations:** a University of Iowa, Department of Pharmacy Practice and Science, 180 S Grand Avenue 339 CPB, Iowa City, IA 52242, United States of America; b CPESN IOWA, 711 55th St, Des Moines, IA 50312, United States of America

**Keywords:** Community pharmacy, Hypertension, Pharmacy practice transformation

## Abstract

**Objectives:**

To implement the Cardiovascular Practice Transformation (CPT) program and evaluate its impact on blood pressure, and to assess the feasibility of implementing the CPT program by identifying obstacles and facilitators.

**Methods:**

Twenty-three Iowa pharmacies participated in the program, each monitoring approximately 10 hypertensive patients for 6 months. Pharmacists assessed blood pressure, medication adherence and addressed medication-related problems during patient visits. Pharmacists used a JotForm application to report patient demographics and care provided during visits. Also, blood pressure readings were collected or measured and reported. After six months, an online Qualtrics survey was sent to participating pharmacies to assess obstacles and facilitators to CPT program implementation. Descriptive statistics were calculated for the patient sample and paired *t*-tests compared baseline and final blood pressure readings. Frequencies were calculated for obstacles and facilitators.

**Results:**

A total of 232 patients participated, with 138 patients having both baseline and follow-up blood pressure data. Systolic blood pressure decreased from 144.2 to 133.6 mmHg, and diastolic blood pressure decreased from 84.4 to 78.3 mmHg, with *p* < 0.01 for both. Peer coaching and CPT resources were the main facilitators, while obstacles included documentation and staff time constraints.

**Conclusion:**

Overall, the CPT program successfully supported pharmacies in improving blood pressure management.

## Highlights

• The Cardiovascular Practice Transformation (CPT) program supported pharmacists in monitoring and lowering blood pressure.
• Key obstacles to changing practices to monitor blood pressure were: reimbursement, staff time, and documentation.
• Peer coaching and CPT resources were the main facilitators for successfully monitoring and managing blood pressure.

## 1 Introduction

Hypertension is a prevalent global disease and a major risk factor for cardiovascular disease. In the United States, 48.1 % of the adult population has hypertension.^1^ Uncontrolled hypertension can lead to substantial cardiovascular complications.2., 3, 4. These complications significantly affect the patient's quality of life (QoL) and contribute to the rise in healthcare expenditures.2., 3, 4. Previous studies highlight the impact of pharmacists on achieving controlled hypertension.^5^^,^^6^ A key question is how to translate these research findings into practical applications in community pharmacies.

Community pharmacies have been working to transform their practices from a focus on dispensing medications to a broader perspective that includes medication management services. To help community pharmacies to transform their practices, Flip the Pharmacy (FTP) has emerged as an initiative actively embraced by members of the Community Pharmacy Enhanced Services Network (CPESN) USA.^7^ A key purpose of FTP is to transform community pharmacy practices to better deliver care services for patients managing chronic conditions such as hypertension and diabetes.

Over the past three years, CPESN Iowa, a member of CPESN USA, has successfully participated in the Flip the Pharmacy (FTP) program. Building on this success, the Cardiovascular Practice Transformation (CPT) program, a collaboration between the University of Iowa, the Iowa Department of Public Health and CPESN Iowa, was developed as a modified approach of FTP to guide pharmacies caring for patients with cardiovascular conditions such as hypertension. The CPT program provides peer coaching and materials to support longitudinal monitoring of patients with hypertension (HTN), addressing five domains for practice transformation. By integrating these change packages into their practices, participating community pharmacies were guided in their application of transformative strategies for monitoring and managing blood pressure.

The objectives of this project were: (1) to implement the CPT program and evaluate its impact on blood pressure and (2) to assess the feasibility of implementing the CPT program by identifying obstacles and facilitators.

## 2 Materials and methods

For this project, 23 CPESN Iowa pharmacies participated in the (CPT) program. The components of the CPT program were peer coaching of pharmacists, monthly online change packages guiding practice transformation and performance feedback through a project dashboard. The change packages addressed five domains of Flip the Pharmacy (www.flipthepharmacy.com): 1) Leveraging the Appointment-Based Model (medication synchronization), 2) Improving Patient Follow-up and Monitoring, 3) Developing New Roles for Non-Pharmacist Support Staff, 4) Optimizing the Utilization of Technology and eCare Plans and 5) Establishing Working Relationships with Other Care Team Members. Previous Flip the Pharmacy change packages were modified to focus on longitudinally monitoring patients with hypertension. Participating pharmacists were encouraged to use a self-measured blood pressure (SMBP) approach for patients willing and able to do so. The self-measured blood pressure (SMBP) program consisted of three main steps: (1) Screen patients for the ability and necessity of home blood pressure monitoring, (2) Train patients to collect and report home blood pressure readings, and (3) Coordinate care with healthcare providers and patients to manage blood pressure effectively. The program utilized validated automatic digital blood pressure monitors approved by the American Medical Association (AMA) and listed on the ValidateBP.org database. Patients were instructed to take 12 readings over 3–7 days, with two readings each morning and evening, one minute apart in the same sitting, while maintaining proper seated positioning and arm placement. Pharmacists conducted training sessions to provide instructions on preparation steps (e.g., avoiding caffeine, exercise, or smoking 30 min prior), proper device usage, and reporting approaches. Results were documented, reviewed, and averaged by pharmacists, who collaborated with healthcare providers to assess trends and adjust treatment as necessary. This structured approach ensured consistent measurement techniques and reliable outcomes across participating pharmacies.

Each of the five change packages contained advice about a particular domain. They were emailed to the participating pharmacy contacts and made available on a project web site. Change Package 1 described how to approach medication synchronization and included a medication synchronization self-assessment. Also, the change packages followed a sample patient case that gradually added issues and illustrated ways to document related care activities. Change Package 2 focused on improving patient follow-up and monitoring. It included a follow-up guide for monitoring patients with HTN. Change Package 3 addressed developing new roles for support staff and included discussions of involving the whole pharmacy team in monitoring and workflow change examples. Change Package 4 utilized a podcast to discuss use of technology to support MTM services and practice transformation. It also included a list of best practices for technology use. Change Package 5 focused on establishing working relationships with other providers. It raised issues about care coordination, communication and sharing information about mutual patients.

The change packages were supported in the CPT program by regular (e.g., monthly) pharmacist interactions with peer coaches, either face-to-face or virtually. Theses coaches, who typically were experienced with progressive community pharmacy practice, worked with their assigned pharmacies to determine how to implement the key components of the change packages. Sometimes the coach led a discussion of changes in workflow at the pharmacy. Other times the coach could provide resources about a concept or change raised in a change package. Such resources could include their own expertise, written materials and connections with other pharmacists who had resolved a similar issue. The peer coaches were an important part of the CPT program.

This project used a pre-post design where each pharmacy was asked to monitor 10 patients with hypertension and/or diabetes for up to six months. During CPT visits with patients, the pharmacists checked blood pressure, assessed medication adherence, identified medication-related problems (MRPs) and took action to address the identified problems. Pharmacists reported their CPT activities using a project JotForm application (electronic data collection tool).

The JotForm collected two types of reports: baseline and follow-up. The baseline report consisted of items designed to collect patient-specific information during the initial visit. These items included the Patient Identification Number (a unique ID), pharmacy name and National Provider Identifier (NPI), date of service, social determinants of health screenings, current disease states being treated, statin usage, and adherence issues. Additionally, pharmacists reported the number of chronic medications taken monthly, identified medication therapy problems (MTPs), and noted enrollment in MedSync programs. Pharmacists could also provide narrative details regarding patient success stories, challenges, or social determinants of health findings while ensuring no Protected Health Information (PHI) was shared. The form was structured to streamline data collection.

The follow-up reports documented care activities and additional patient information obtained during subsequent visits. These included updates on blood pressure readings, medication usage, and interventions addressing MTPs. Follow-up reports also allowed pharmacists to track patient progress, share observations, and collaborate with healthcare providers to adjust treatment plans as needed.

For analyses of JotForm data, descriptive statistics were calculated for all patients in the sample. For patients with multiple blood pressure readings, baseline and the last follow-up blood pressure readings were compared using paired *t*-tests.

After six months, participating pharmacies received an online Qualtrics survey to assess obstacles and facilitators to the CPT program's implementation (**Provo, UT)**. Pharmacists classified 13 items as either facilitators or obstacles, and frequencies were calculated for each.

This project was reviewed and judged to be exempt by the University of Iowa Institutional Review Board (IRB).

## 3 Results

The baseline data included 232 patients from 23 pharmacists, with a mean age of 62.4 years and a median age of 63 (range: 21–92) (Table 1). Of these patients, 53.4 % were males and 92.7 % identified as Caucasian or White. On average, these patients were prescribed 7 chronic medications per month with a median of 6 (range: 0–60).

**Table 1:** Characteristics of Patients Receiving Care under CPT Program.

Characteristic	Frequency (%)
Gender	
Female	108 (46.6)
Male	124 (53.4)
Race	
Caucasian or white	215 (92.7)
Other race	5 (2.2)
Unknown or missing	12 (5.1)
Insurance type	
Medicare only	100 (43.1)
Commercial	80 (34.5)
Medicaid only	36 (15.5)
Medicare and Medicaid	10 (4.3)
Other insurance	4 (1.7)
Uninsured	2 (0.9)
Disease state	
Hypertension	197 (84.9)
Patient is enrolled in medication synchronization	136 (58.6)
Patient with HTN enrolled in self-measured BP program^A^	69 (35.0)
Smoking status (yes)	26 (11.2)


	Mean (SD)
Age (years)	62.4 (14.0)
Number of chronic medications taken monthly	7.0 (5.3)

A Denominator of percent was number of patients with HTN (*n* = 197).

Out of 197 patients diagnosed with hypertension, 138 had both baseline and follow-up measurements and were included in the blood pressure analyses. Both systolic and diastolic blood pressure readings decreased significantly (*p* < 0.01) from baseline to the last follow-up BP reading. Mean (SD) systolic blood pressure decreased from 144.2 (21.3) mm Hg to 133.6 (18.5) mm Hg, while mean diastolic blood pressure decreased from 84.4 (13.2) to 78.3 (11.3) mm Hg.

The most reported facilitators of the CPT program among participating pharmacies were change packages and coaching (87 %, *n* = 20), followed by the dashboard (78.3 %, *n* = 18) and the web-based form tool (73.9 %, *n* = 17). The most frequently cited obstacles were service documentation (73.9 %, n = 17), staff time (69.6 %, *n* = 16), and lack of reimbursement (65.2 %, *n* = 15) (Table 2).

**Table 2:** Facilitators and Obstacles for Cardiovascular MTM (*N* = 23).

Practice Factor	Facilitator Frequency (%)	Obstacle Frequency (%)
CPT change packages	20 (87.0)	3 (13.0)
CPT peer coaching	20 (87.0)	3 (13.0)
CPT Dashboard	18 (78.3)	5 (21.7)
CPT Jot Form	17 (73.9)	6 (26.1)
Access to patient information	14 (60.9)	9 (39.1)
Communication with providers	14 (60.9)	9 (39.1)
Patient engagement in their care	13 (56.5)	10 (43.5)
Provider response to your MTM activities	12 (52.2)	11 (47.8)
Staff engagement in the care^A^	11 (50.0)	11 (50.0)
Changes in workflow	10 (43.5)	13 (56.5)
Reimbursement for services	8 (34.8)	15 (65.2)
Staff time	7 (30.4)	16 (69.6)
Documentation of services	6 (26.1)	17 (73.9)

A Responses do not total 23 due to missing data.

## 4 Discussion and conclusions

The CPT program had a positive impact on the monitoring and management of patients with uncontrolled blood pressure. By the end of the study, the mean blood pressure fell below 134/79 mmHg. These blood pressure levels achieved targets for patients, according to hypertension treatment guidelines.^8^ The findings here are consistent with previous studies showing community pharmacists can help improve blood pressure levels. A randomized control study involving over 50 community pharmacies reported a significant reduction in the risk for cardiovascular events due to a reduction in blood pressure.^9^ Also, a collaborative model between an academic medical center and community pharmacies showed higher blood pressure control rates compared to usual care.^10^ A meta-analysis of 16 RCTs involving community pharmacist-led interventions for patient with hypertensions showed significant reductions in both systolic and diastolic blood pressure.^11^ The findings of this study add to the evidence that community pharmacists can improve control of hypertension when given the resources and guidance to do so.

The model used in the CPT program was derived from the Flip the Pharmacy program. The CPT program addressed medication synchronization, patient follow-up and monitoring, development of new roles for non-pharmacist staff members, utilization of technology and connection with other care team members. The CPT program supports pharmacists in delivering longitudinal blood pressure monitoring, refining their SMBP program and coordinating care with local providers, as well as helping them connect with local providers if they haven't already done so. Furthermore, the program works with participating practices to establish workflows that support the delivery and documentation of MTM services, and training the staff in using technology for documentation, improving patient outcomes and ensuring comprehensive care. In future research, a promising approach to study the effect of CPT program on community pharmacy practice transformation is to adopt the Systems Engineering Initiative for Patient Safety (SEIPS) model to better understand changes, obstacles and facilitators. The SEIPS model involves a work system that includes domains of people, tasks, tools and technology, organization and environment.^12^

There were three major perceived obstacles reported by pharmacists: financial obstacles, documentation of services and staff time. First, financial obstacles, such as low payment for dispensing and lack of payment for disease state management, present significant challenges for pharmacies, limiting resources and potentially reducing staffing levels due to decreased revenue. However, these obstacles also underscore the pressing need for pharmacies to develop new revenue streams, with services like longitudinal monitoring emerging as promising avenues. As noted by Nguyen et al.,^13^ some states have implemented Medicaid programs that pay pharmacists for medication and/or disease state management services. To support more pharmacists delivering hypertension management services, more states could incorporate similar approaches into their Medicaid programs. Another suggestion is to convey Federal provider status upon pharmacists, which would allow expansion of Medicare reimbursement programs to include pharmacist services under Medicare Part B.^14^ Additionally, establishing collaborative practice agreements can open opportunities for pharmacists' payment and enhance the role of pharmacists in improving healthcare outcomes. In commenting on return on investment the U.S. Assistant Surgeon General stated, ‘It has been studied and noted that for every $1 spent on pharmacist services, $10 are saved.^15^ Perhaps, now is time to actively expand payment for pharmacist services.

Second, documenting services and the need to develop and refine workflows present significant obstacles. Pharmacists often face challenges in integrating new documentation requirements into their existing workflows. The time-consuming nature of thorough documentation can be a challenge, especially in busy pharmacy settings where pharmacists are already managing high volumes of tasks. These obstacles could be addressed through investment in user-friendly documentation systems that are integrated seamlessly into pharmacy operations.^16^ Training pharmacists on efficient documentation practices and utilizing technology can streamline this process, making it more manageable and less time-consuming. The CPT program worked with participating practices to establish workflows that support the delivery and documentation of services, as well as training the staff in using technology for documentation.

The third obstacle was staff time. Limited staff or inefficient workflows can lead to pharmacists spending insufficient time delivering services. Our finding is consistent with what has been reported in the literature about time constraints.^17^ A solution for this issue could be changing the workflow and improving time management.^18^ For example, some of the participating community pharmacies were able to have pharmacy technicians handle the distributional activities of medication dispensing, allowing pharmacists more time to engage clinically with patients. Establishing new services often involves trial and error. The CPT program addressed this by helping pharmacists increase the use of Med Sync, which has been shown to reduce time spent on dispensing tasks, potentially freeing up pharmacists to deliver other services.^19^

There are several limitations to consider within this project. First, the pharmacy sample limits the generalizability of the results. Supported by the CPT program, the participating pharmacists were able to work with their targeted patients. However, it is not clear how well pharmacists in other community pharmacies would be able to work with their patients with hypertension. Future research could be done to compare the effects of community pharmacies that had transformed their practices with pharmacies that have not done so. Second, employing a pre-post quasi-experimental design without control arms and randomization may introduce systematic biases into the outcomes. Without randomization, patients may differ in ways that could influence the results. Perhaps future work with implementation designs could be used to systematically address validity threats.

In conclusion, the CPT program represents a valuable initiative in enhancing pharmacy practice and improving patient outcomes in managing uncontrolled blood pressure.

## Funding

This work was supported by a grant from the 10.13039/100008944Iowa Department of Public Health.

## CRediT authorship contribution statement

**William R. Doucette:** Writing – review & editing, Writing – original draft, Validation, Supervision, Project administration, Methodology, Investigation, Funding acquisition, Formal analysis, Data curation, Conceptualization. **Eilan Alhersh:** Writing – review & editing, Writing – original draft, Formal analysis. **Lindsey Ludwig:** Writing – review & editing, Resources, Methodology, Investigation, Funding acquisition, Conceptualization. **Stevie Veach:** Writing – review & editing, Resources, Methodology, Investigation, Funding acquisition, Conceptualization.

## Declaration of competing interest

The authors declare that they have no known competing financial interests or personal relationships that could have appeared to influence the work reported in this paper.
